# Regeneration of highland papaya (*Vasconcellea pubescens*) from anther culture

**DOI:** 10.1002/aps3.1182

**Published:** 2018-09-24

**Authors:** Borys Chong‐Pérez, Basilio Carrasco, Herman Silva, Francisca Herrera, Karla Quiroz, Rolando Garcia‐Gonzales

**Affiliations:** ^1^ Sociedad de Investigación y Servicios BioTECNOS Ltda. Camino a Pangal, Km 2.5 San Javier Chile; ^2^ Departamento de Ciencias Vegetales Facultad de Agronomía e Ingeniería Forestal Pontificia Universidad Católica de Chile Av. Vicuña Mackenna 4860 Santiago Chile; ^3^ Laboratorio de Genómica Funcional & Bioinformática, Facultad de Ciencias Agronómicas Universidad de Chile Av. Santa Rosa 11315, 8820808 La Pintana Santiago Chile; ^4^ Centro de Biotecnología de los Recursos Naturales Facultad de Ciencias Agrarias y Forestales Universidad Católica del Maule Avenida San Miguel 3605 Talca Chile

**Keywords:** callus, Caricaceae, in vitro culture, plant regeneration, somatic embryos, *Vasconcellea pubescens*

## Abstract

**Premise of the Study:**

*Vasconcellea pubescens* is an important Caricaceae species cultivated in several countries of South America. The objective of this study was to investigate different media compositions and plant growth regulators to induce plant regeneration.

**Methods:**

Anthers were cultured in Murashige and Skoog medium with varying concentrations of naphthalene acetic acid (NAA) and 2,4‐dichlorophenoxyacetic acid (2,4‐D) plus a cytokinin (N‐(2‐chloro‐4‐pyridyl)‐N′‐phenylurea). The effect of the basal medium supplemented with auxins and cytokinins on shoot regeneration from the induced calli was also evaluated. Addition of maltose to the basal medium was also tested.

**Results:**

The combination of 0.54 μM NAA and 22.66 μM 2,4‐D induced the highest rate of calli formation. Regeneration via organogenesis was obtained in Murashige and Skoog and Woody Plant Medium supplemented with maltose and containing 8.88 μM 6‐benzylaminopurine, 5.71 μM indoleacetic acid, and 2.28 μM zeatin.

**Discussion:**

The plant regeneration protocol reported here permits the development of haploid and double haploid plants that can be useful for propagation purposes, allow a better molecular understanding of the species, and facilitate the production of new cultivars.

The Caricaceae family comprises approximately 35 species in six genera (*Carica* L., *Vasconcellea* A. St.‐Hil., *Jacaratia* A. DC., *Cylicomorpha* Urb., *Papaya* Mill., *Jarilla* Rusby). Alongside *Carica papaya* L., the most popular representative of the family, *V. pubescens* A. DC. (also known as *V. cundinamarcensis* V. M. Badillo) is a fruit plant that has become increasingly important in South America, especially in Chile. This species is found in tropical regions, mainly in South America, at altitudes above 1500 m a.s.l., and it is native to the Ecuadorian Andes (National Research Council, 1989; Badillo, 2000; Van Droogenbroeck et al., 2002; Morales et al., 2004; Carrasco et al., 2009; d'Eeckenbrugge et al., 2014). It is usually processed to produce conserved fruit, juice, jam, and sweets. Moreover, *V. pubescens* plants produce latex with a high level of papain, an important and valuable proteolytic enzyme that is used in the health and cosmetics industries, and also has other applications in softening textiles, silk, and leather; as a meat tenderizer; and in beer production (Carrasco et al., 2009, 2014).

In Chile, the genetic diversity of *V. pubescens* is affected by its culture conditions, in which every farmer selects the best plants and collects the seeds from their fruits to produce seedlings (Carrasco et al., 2009). To date, several problems have been associated with such agronomic practices, the most important being the scarcity of cultivars and plant materials with high phytosanitary quality. Carrasco et al. (2009) proposed a national breeding program to increase the genetic diversity of this plant by crossing material from different cultivated areas. In addition, hermaphroditic plants have now been found, which makes it possible to pollinate female plants without the need for male plants (Salvatierra‐González and Jana‐Ayala, 2016).

Breeding programs need homozygous plants that are obtained by several generations of self‐pollination, which is a time‐consuming process, especially in fruit species such as *V. pubescens*. One way to overcome this problem is through the use of biotechnological tools to generate haploid and double haploid lines (Forster et al., 2007). Currently, the most effective methods to obtain haploid and double haploid materials are in vitro cultures of anthers or isolated microspores (called androgenesis), and less commonly, ovary culture (gynogenesis) (Germanà, 2011). Although haploid and double haploid plants have been obtained for many crop plants (reviewed in Maluszynski et al., 2003; Germanà, 2011), this has not yet been done for *Vasconcellea* species. However, in tissue culture, many of the protocols developed for a single species can be transferable to other relatives within genera and families (Magdalita et al., 1996). In the Caricaceae, there are only a few reports for *C. papaya* being used to produce haploid and double haploid plants, and all have used anther culture (Litz and Conover, 1978, 1979; Tsay and Su, 1985; Rimberia et al., 2005). For this related species, the addition of naphthalene acetic acid (NAA) and N‐(2‐chloro‐4‐pyridyl)‐N′‐phenylurea (CPPU) produced 13.8% of embryo induction when the anthers were cultivated on Murashige and Skoog (MS; Murashige and Skoog, 1962) basal medium. In the study by Rimberia et al. (2006), rooting of the germinated embryos was obtained by putting the basal part of the plants in a 50% ethanol solution containing 1500 mg·L^−1^ of indole‐3‐butyric acid (IBA); all of the resulting plants were female. Furthermore, in a later study, Rimberia et al. (2007) found that the plants produced from anther culture were phenotypically different, with variable fruit yields. Rimberia et al. (2007) proposed that these plants had considerable potential for commercial exploitation and for use in breeding programs. These observations could have practical implications because haploid or double haploid plants generated by anther culture could produce fruit under orchard conditions.

The production of haploid or double haploid plants in *V. pubescens* has not previously been reported. Jordan and Velozo (1997) reported the formation of somatic embryos from anthers cultured with NAA and 6‐benzylaminopurine (6‐BAP), but these authors did not discuss the regeneration of plants from these embryos.

In this study, we determined a series of key parameters necessary to obtain plants of *V. pubescens* through anther culture, as a first stage in the production of haploid and double haploid plants. These included the appropriate developmental stage to excise and culture the microspore‐carrying anthers, the type and concentration of plant growth regulators, and the carbon source.

## MATERIALS AND METHODS

### Plant material and pollen developmental stage

Male flowers (10–30 mm) were collected from 26 individuals of four‐ to five‐year‐old field‐grown monoecious plants from September to November 2016, in the locality of Lipimávida, Región del Maule, Chile (34°50′48.9″S, 72°08′30.8″W). Flowers were placed in four groups according to their size: I = 10–15 mm, II = 15–20 mm, III = 20–25 mm, and IV = ≥25 mm. Only flowers from group IV were fully open (Table 1).

**Table 1:** Microspore/pollen developmental stage distributions in anthers of different flower size classes in *Vasconcellea pubescens*

Group (Size)	Tetrad (%)	Uninucleate (%)	Early binucleate pollen (%)	Late binucleate pollen (%)	Mature pollen (%)
I (10–15 mm)	23.8	76.2	—	—	—
II (15–20 mm)	4.2	80.3	15.5	—	—
III (20–25 mm)	1.3	23.2	66.2	9.3	—
IV (≥25 mm)	—	—	—	5.4	94.6

Five anthers from five flowers per size group were used for microscopic analysis. Anthers from flowers from different size groups were macerated in 1.5‐mL tubes, fixed in a 3 : 1 ethanol : glacial acetic acid mixture for 10 min, rinsed with distilled water, and stained with a few drops of 4′,6‐diamidine‐2‐phenylindole dihydrochloride (DAPI) solution (0.1 M sodium phosphate [pH 7.0], 1 mM EDTA, 0.1% Triton X‐100, 0.4 μg/mL DAPI). The stained microspores were observed under a fluorescence microscope (FLoid Cell Imaging Station, Life Technologies, Thermo Fisher Scientific, Waltham, Massachusetts, USA) to determine the pollen developmental stage and the number of pollen grains in each stage.

Flowers belonging to groups that showed the highest percentage of microspores in uninucleate/vacuolated and early binucleate stages were selected as initial plant material. Selected flowers were immersed in 500 mg·L^−1^ CuSO_4_ sterile solution for four days at 4°C, with the aim to avoid fungal contamination and to improve anther viability (Brew‐Appiah et al., 2013). Flowers were surface‐sterilized by immersion in 70% (v/v) ethanol for 15 min, washed three times in sterile distilled water, and submerged in 1% (w/v) NaOCl for 15 min. Finally, the flowers were rinsed three times in sterile distilled water. Then, the anthers were isolated from the flowers and placed in a 54.65 g·L^−1^ mannitol solution until their inoculation onto the induction medium. MS basal medium and several combinations of growth regulators (2,4‐dichlorophenoxyacetic acid [2,4‐D]: 0, 11.33, 22.66, or 33.99 μM; NAA: 0 or 0.54 μM; and CPPU: 0, 0.005, 0.01, 0.02, or 0.04 μM) were tested for callus induction (Table 2). Sucrose (20 g·L^−1^) was added in all cases, and the pH of the medium was adjusted to 5.8 prior to gelling with 7.5 g·L^−1^ agar (Tecnologia y Ciencia, Región Metropolitana, Chile) and autoclaving. Ten anthers were cultured in separate glass jars (130‐mL capacity) containing 15 mL of culture medium. The cultures were maintained in the dark at a temperature of 25 ± 2°C for two months.

**Table 2:** Regeneration media compositions used for morphogenesis induction in anthers of *Vasconcellea pubescens*.^a^

Component	MSS	MSM	WPS	WPM	WPG	WPWR
MS salts (%)	100	100	—	—	—	—
MS vitamins (%)	100	100	—	—	—	—
WP salts (%)	—	—	100	100	100	100
WP vitamins (%)	—	—	100	100	100	100
6‐BAP (μM)	8.88	8.88	8.88	8.88	1.2	—
IAA (μM)	5.71	5.71	5.71	5.71	—	—
Zeatin (μM)	2.28	2.28	2.28	2.28	—	—
NAA (μM)	—	—	—	—	1.5	—
Sucrose (g·L^−1^)	30	—	30	—	—	—
Maltose (g·L^−1^)	—	20	—	20	20	20

Note

6‐BAP = 6‐benzylaminopurine; IAA = indoleacetic acid; MS = Murashige and Skoog medium (Murashige and Skoog, 1962); NAA = naphthalene acetic acid; WP = Woody Plant Medium (McCown and Lloyd, 1981); MSS = Murashige and Skoog medium (Murashige and Skoog, 1962) supplemented with 8.88 μM 6‐BAP, 5.71 μM IAA, 2.28 μM zeatin, and 30 g·L^−1^ sucrose; MSM = same as MSS, but with 20 g·L^−1^ maltose instead of sucrose; WPS = Woody Plant Medium supplemented with the same plant growth regulators and carbon source as MSS; WPM = same as WPS, but with 20 g·L^−1^ maltose instead of sucrose; WPG = Woody Plant Medium supplemented with 1.20 μM 6‐BAP, 1.50 μM NAA, and 20 g·L^−1^ maltose; WPWR = Woody Plant Medium without plant growth regulator and with 20 g·L^−1^ maltose.

a The pH of the medium was adjusted to 5.7–5.8 prior to autoclaving, and the medium was gelled with 7.5 g·L^−1^ agar.

The calli that had formed on the anthers were then transferred to 130‐mL glass jars containing 15 mL of multiplication medium (MS basal medium supplemented with 22.66 μM 2,4‐D, 0.54 μM NAA, 2.28 μM zeatin, and 30 g·L^−1^ sucrose). The pH of the medium was adjusted to 5.7–5.8 prior to autoclaving, and the medium was gelled with 7.5 g·L^−1^ agar. The cultures were maintained in a culture room for 28 d, at a temperature of 25 ± 2°C under a 16 h/8 h photoperiod using cool white fluorescent lamps, at a photosynthetic photon flux density of 100 μmol·m^−2^·s^−1^.

### Plant regeneration from anther callus

Plant regeneration was first performed using a 250‐mL glass jar containing 25 mL of MS medium supplemented with sucrose (MSS medium; Table 2). These cultures were maintained at the same conditions as previously detailed for calli culture. Next, based on the study by Rimberia et al. (2006), callus regeneration was tested on MS and Woody Plant Medium (WP; McCown and Lloyd, 1981) supplemented with different combinations of plant growth regulators and carbon sources. For these experiments, calli were transferred to glass flasks containing medium (~1 g of fresh mass per flask), and the cultures were maintained at the same conditions previously detailed for plant regeneration and calli culture. At least five flasks were used for each treatment. The regenerated shoots were transferred to a modified elongation culture medium proposed by Posada‐Pérez et al. (2007) for *C. papaya* (100% MS salts supplemented with 1 mg·L^−1^ thiamine HCl, 1.2 μM 6‐BAP, 1.5 μM NAA, 100 mg·L^−1^ myo‐inositol, 30 g·L^−1^ sucrose, 1.0 μM riboflavin, and 7.5 g·L^−1^ agar; pH adjusted to 5.8 before autoclaving).

### Statistical analysis

For shoot regeneration, at least five replicates were assessed in each experiment (the total number of calli cultivated in all replicates ranged from 15 to 42). The number of shoots per flask after 28 d was compared by a nonparametric Kruskal–Wallis test using SPSS version 21 (SPSS Inc., Chicago, Illinois, USA). This test was used because the data violated necessary assumptions for parametric testing and transformation did not offer significant improvement. To distinguish between comparisons, a post hoc Mann–Whitney test was performed. Significance was reported at *P* ≤ 0.05.

## RESULTS

**Figure 1:**
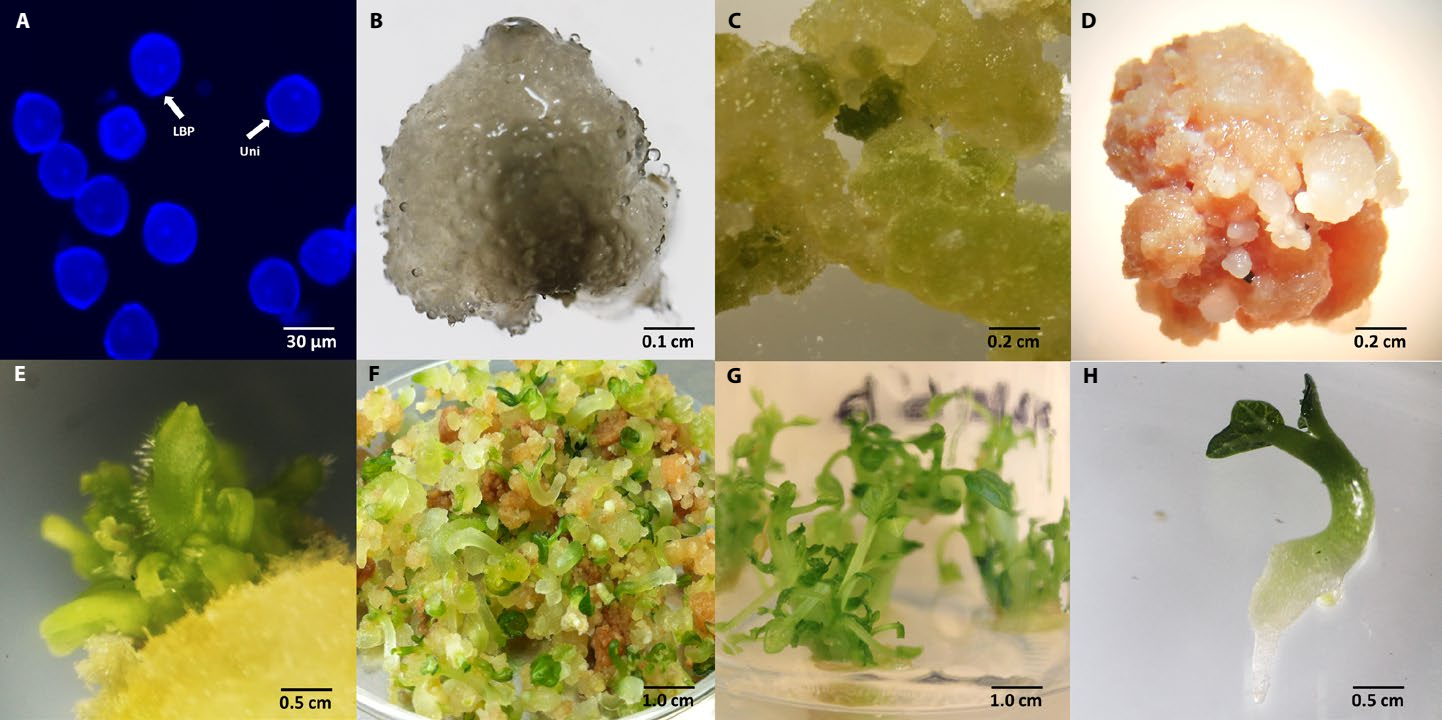
Shoot regeneration from anther culture in *Vasconcellea pubescens*. (A) Microspore/pollen developmental stage. (B) Calli induction in *V. pubescens* anther after one month of culture. (C) Development of green callus. (D–F) Regeneration through organogenesis in *V. pubescens* anther‐derived callus. (G) Completely differentiated shoots. (H) *V. pubescens* anther‐derived plants cultured on elongation culture medium. Uni = uninucleate microspore, LBP = late binucleate pollen.

A primary DAPI analysis performed to study the correlation between flower bud size and pollen developmental stage was used to select the plant material to initiate the cultures. The DAPI analysis showed that flowers between 15–25 mm (groups II and III) had more than 90% of microspores in the uninucleate/vacuolated and early binucleate stages (Table 1, Fig. 1A). Thus, flower buds ranging from 15–25 mm (closed flowers) were selected for further experiments.

### Callus induction

Fifteen days after inoculation in MS medium containing different combinations of growth regulators, some anthers exhibited swelling and rupture of the microspore sac. The rest of the anthers became darker in color or did not show any response. After a month of culture in dark conditions, calli were visible in the swelling anthers (Fig. 1B). In general, the rate of callus induction was very low (Table 3). The combinations of 2,4‐D and NAA were the most effective for callus induction (Table 3). The highest rate (10%) was obtained in medium containing 22.66 μM of 2,4‐D and 0.54 μM of NAA. Calli were also induced in CPPU‐containing medium, but at a lower frequency, but the combination of 2,4‐D, CPPU, and NAA inhibited the formation of calli (Table 3).

**Table 3:** Effects of 2,4‐D, NAA, and CPPU on callus induction in *Vasconcellea pubescens* anther after one month of culture

Treatment no.	2,4‐D (μM)	NAA (μM)	CPPU (μM)	No. of anthers forming calli (%)^a^
1	0	0	0	2 (1.43)
2	11.33	0	0	4 (2.86)
3	11.33	0.54	0	9 (6.43)
4	22.66	0	0	4 (2.86)
5	22.66	0.54	0	14 (10.00)
6	33.99	0	0	5 (3.57)
7	33.99	0.54	0	6 (4.28)
8	0	0.54	0.02	3 (2.14)
9	0	0.54	0.04	5 (3.57)
10	0	5.43	0.04	1 (0.71)
11	2.5	1.0	0.005	2 (1.43)
12	5.0	0.1	0.005	2 (1.43)
13	5.0	1.0	0.005	1 (0.71)
14	2.5	0.1	0.01	0 (0.00)
15	2.5	1.0	0.01	3 (2.14)
16	5.0	0.1	0.01	0 (0.00)
17	5.0	1.0	0.01	0 (0.00)

Note

2,4‐D = 2,4‐dichlorophenoxyacetic acid; CPPU = N‐(2‐chloro‐4‐pyridyl)‐N′‐phenylurea; NAA = naphthalene acetic acid.

a A total of 140 anthers were used in each treatment.

### Callus proliferation and shoot regeneration

In vitro–produced calli retained the ability to further multiply when transferred to the multiplication medium. After two months, calli were transferred to a regeneration medium containing 6‐BAP, zeatin, and indoleacetic acid (IAA) as detailed in Table 2. Two weeks later, the morphology of the calli changed: some calli turned green, while other calli showed compact structures (Fig. 1C). These changes were independent of treatment where the callus originated and were visible in all callus lines. In this medium, calli continued their growth and multiplication.

Shoot regeneration via organogenesis occurred in 100% of the calli 45 d after they were transferred to regeneration medium (Fig. 1D). After several subcultures in the MSS medium, some calli continued to regenerate by organogenesis at a very low rate (1–2 shoots per flask).

Moreover, there were three callus lines, one from treatment 2 and two from treatment 5, that showed the formation of creamy white, round, compact, and independent structures (disconnected from the mother callus) that resembled somatic embryos (Fig. 1E). These structures were able to produce shoots, even as most of them continued with secondary multiplication (Fig. 1F). Regeneration from these calli was then tested in several MS or WP basal medium mixtures.

**Figure 2:**
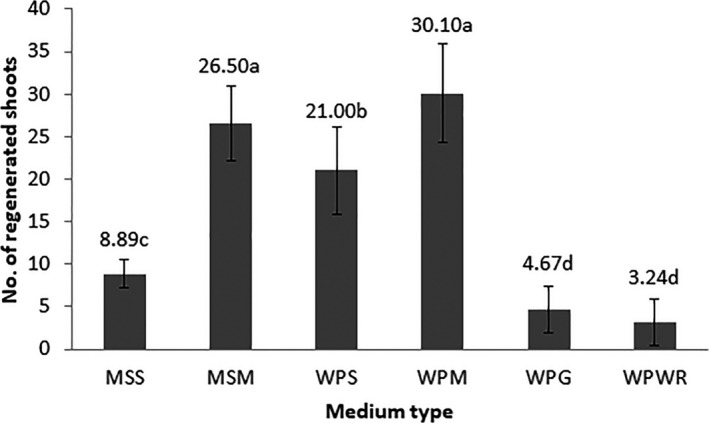
Effects of culture medium, carbon source, and plant growth regulators on shoot regeneration from anther‐derived calli in *Vasconcellea pubescens*. The pH of the medium was adjusted to 5.7–5.8 prior to autoclaving, and the medium was gelled with 7.5 g·L^−1^ agar. Different letters represent significant differences between treatments according to Kruskal–Wallis analysis (*P* ≤ 0.05). MSS = Murashige and Skoog medium (Murashige and Skoog, 1962) supplemented with 8.88 μM 6‐BAP, 5.71 μM IAA, 2.28 μM zeatin, and 30 g·L^−1^ sucrose; MSM = same as MSS, but with 20 g·L^−1^ maltose instead of sucrose; WPS = Woody Plant Medium (McCown and Lloyd, 1981) supplemented with the same plant growth regulators and carbon source as MSS; WPM = same as WPS, but with 20 g·L^−1^ maltose instead of sucrose; WPG = Woody Plant Medium supplemented with 1.20 μM 6‐BAP, 1.50 μM NAA, and 20 g·L^−1^ maltose; WPWR = Woody Plant Medium without plant growth regulator and with 20 g·L^−1^ maltose.

As shown in Figure 2, WP and MS media containing maltose as the carbon source and a combination of 8.88 μM 6‐BAP, 5.71 μM IAA, and 2.28 μM zeatin (WPM and MSM, respectively) were the most effective for shoot regeneration. Calli cultured in WP medium containing sucrose (WPS) and the same concentrations of plant growth regulators showed less efficiency than those cultivated in WPM and MSM (Fig. 2). Nevertheless, when MS medium was combined with the same concentrations of plant growth regulators and sucrose (MSS), there was one third less calli regeneration than in the best treatments (Fig. 2).

Treatment with MSS showed more than a twofold (2.36) reduction in calli regeneration when compared with WPS. Furthermore, some calli produced a few shoots in media containing low amounts of 6‐BAP, NAA, and maltose (WPG) or in media without plant growth regulators and with maltose (WPWR). In total, 832 shoots were obtained (Fig. 1G). Most of these shoots were derived from embryo‐like structures (777 shoots, 93.4% of the total). Remarkably, only 2% of the shoots coming from these structures were able to produce shoots and roots simultaneously (Fig. 1F). In our conditions, most of the regenerated shoots produced calli at the base of the explant, before root emission.

## DISCUSSION

Several factors can influence in vitro androgenesis, including genotype, physiological state and growth conditions of donor plants, season, microspore developmental stage, culture medium, preculture treatments, plant growth regulators, and culture conditions (Rimberia et al., 2005, 2006; Germanà, 2011). The results of the current study established that most microspores were in the uninucleate/vacuolated and early binucleate stages in flower buds of sizes ranging from 15–25 mm. These microspores have been reported as those in the most responsive state to induce the production of embryogenic or organogenic calli in several plant species such as *Citrus clementina* Hort. ex Tan (Germanà and Chiancone, 2003), *C. papaya* (Rimberia et al., 2005), *Olea europaea* L. (Solís et al., 2008), *Brassica napus* L. (Prem et al., 2012), and *Eriobotrya japonica* (Thunb.) Lindl. (Blasco et al., 2015). Similarly, *C. papaya* flower buds ranging between 10 and 14 mm contained a mixture of 48.4% uninucleate, 23.3% mitotic, and 28.4% binucleate microspores (Rimberia et al., 2006).

Plant growth regulators are used for in vitro androgenesis in anther culture, but their effects depend on many factors, such as plant genotype, type and development of tissue, culture conditions or environment, and even endogenous and exogenous concentration or type of plant growth regulator (Moubayidin et al., 2009; Żur et al., 2015). In *V. pubescens*, the best efficiency for inducing somatic embryos from anthers was observed in MS medium supplemented with NAA 5.37 μM and 6‐BAP 4.44 μM (Jordan and Velozo, 1997). In the related species *C. papaya*, the best combination for somatic embryo induction was CPPU 0.04 μM and NAA 0.54 μM, with 13.8% of embryo regeneration (Rimberia et al., 2006); this is the same combination used in treatment 9 in the current study (Table 3). In other tree species, such as *Populus* ×*beijingensis*,* E. japonica*,* Aesculus hippocastanum* L., and *Azadirachta indica* A. Juss., androgenesis from anther culture has been achieved with combinations of auxins and cytokinins (reviewed in Srivastava and Chaturvedi, 2008; Li et al., 2013; Blasco et al., 2015). Nevertheless, in other examples, such as for apples (*Malus domestica* (Suckow) Borkh.), auxin was not necessary (Höfer et al., 1999; Höfer, 2004). In the experiments reported here, only calli were obtained in the conditions detailed above; no somatic embryos were produced.

The regeneration of plants from anther culture in *V. pubescens* has not previously been reported. However, in *C. papaya*, there are some studies that have reported the regeneration of plants through somatic embryogenesis from anther culture (Litz and Conover, 1978, 1979; Tsay and Su, 1985; Rimberia et al., 2005, 2006, 2007; Gyanchand et al., 2015). Here, plant regeneration in *V. pubescens* through indirect organogenesis is reported. It seems that maltose as a carbon source and the combination of plant growth regulators were key factors in the regeneration process. Previously, Jordan and Piwanski (1997) showed that maltose was very important for shoot initiation in babaco (*Vasconcellea* ×*heilbornii* (V. M. Badillo) V. M. Badillo), a hybrid between *V. pubescens* and *V. stipulata* (V. M. Badillo) V. M. Badillo (Lim, 2012), which is consistent with the results of this work. The fact that shoots were able to regenerate from calli on medium containing low amounts or without plant growth regulators (WPG or WPWR, respectively) indicates that apparently there are enough endogenous hormones available to complete the regeneration process. It is well known that regeneration capacity from the callus depends on many factors, including the content of endogenous hormone or the externally applied plant growth regulator (Bhaskaran and Smith, 1990; Guo et al., 2017). However, for the regeneration from anther‐derived calli reported in this work, further experiments are needed to understand the relationship between endogenous hormone content and plant regeneration.

Another important result from our study was that WP medium showed a better response than MS medium in calli regeneration when the carbon source was sucrose (MSS and WPS; Fig. 2). WP medium has been reported as the most suitable for several morphogenetic processes in other species of *Vasconcellea*, such as *V. chilensis* Planch. ex A. DC. (Jordan, 2011), although the author of that study did not obtain plant regeneration from different explants cultured in MS medium. In terms of further applications, the results obtained here for *V. pubescens* could provide a technological basis for mass production of plants, as it was possible to alter the media composition and plant growth regulators to induce plant regeneration from calli obtained from a nontraditional explant source. This information can also be useful for other species in the Caricaceae family.

The phenomenon of callus formation at the base of the shoots is very common in papayas, and it inhibits the complete germination of somatic embryos. Indeed, it has been previously reported in babaco (Vega de Rojas and Kitto, 1991) and *C. papaya* (Ascencio‐Cabral et al., 2008; Sekeli et al., 2013; Dhekney et al., 2016; Posada‐Pérez et al., 2017). Recently, Posada‐Pérez et al. (2017) circumvented this problem in the germination of *C. papaya* somatic embryos using two strategies: first by adding 475.8 μM phloroglucinol in semisolid medium, and second by cultivating the somatic embryos in liquid medium in a RITA temporary immersion system combining 0.02 μM 6‐BAP and 2.90 μM gibberellic acid. In the future, more research is needed in *V. pubescens* to determine if somatic embryogenesis is occurring and to increase regeneration efficiency.

The Caricaceae family contains a number of important plant species with very high economic and environmental value (Scheldeman et al., 2011; Ming et al., 2012). Many studies are now being conducted on the genetic improvement and conservation (Carrasco et al., 2009, 2014; Carvalho and Renner, 2012; de Oliveira et al., 2012; Brown et al., 2012), genomics (Ming et al., 2008, 2012), and plant propagation (Jordan, 2011; Dhekney et al., 2016; Posada‐Pérez et al., 2017) of many species in this family. The conditions for plant regeneration reported here can be very helpful for some of these projects, as well as for new research line developments.

These results can also assist conservation efforts for species in this plant family, because they increase the availability of species that are difficult to propagate, such as *V. chilensis* (Carrasco et al., 2014), by providing an alternative propagation technique using indirect regeneration.

Haploid and double haploid plants are valuable tools for plant improvement. Completely homozygous double haploid plants can be achieved in one generation, which makes this approach a fast method to produce homozygous lines and to discard individuals expressing strong inbreeding depression (Chen et al., 2011; Murovec and Bohanec, 2012). In contrast, in a conventional breeding program, homozygous lines are developed after at least eight generations of self‐pollination, and normally it is not possible to reach 100% homozygosity (Germanà, 2006).

For these reasons, many efforts have been made to develop haploid and double haploid plants, although the most important advances for cultivar development have been achieved in *Brassica*, barley, maize, rice, rye, and wheat (Maluszynski et al., 2003). According to Germanà (2006), more than 280 cultivars have been produced using various double haploid methods; the majority of these have been produced through anther culture. In this regard, *C. papaya* is a polygamous species, where is possible to identify male, female, and hermaphrodite plants. The anther culture technique has shown to be useful for genetic improvement of female papaya plants because the majority of produced double haploid plantlets are female (Rimberia et al., 2005).

The integration of genomic tools with haploid and double haploid plants provides new opportunities for improving selection methods, maximizing selection gains, and accelerating cultivar development, thus leading to the early release of cultivars with improvements in desirable traits (Dwivedi et al., 2015). This is the first report of the production of *V. pubescens* plants from anther culture, and our results contribute to the development of papaya breeding. In total, more than 800 shoots were obtained (Fig. 1F). The results of this work show that it is possible to obtain plants from anther culture through indirect regeneration. Further investigations are needed to assess the ploidy of the regenerated plants and thereafter to select the most interesting plants for genetic studies and papaya breeding programs, as well as to study the agronomic characteristics of the regenerated plants.
